# Future Trends in *Obolodiplosis robiniae* Distribution across Eurasian Continent under Global Climate Change

**DOI:** 10.3390/insects14010048

**Published:** 2023-01-03

**Authors:** Jia-Qiang Zhao, Tai Gao, Jing-Jing Du, Juan Shi

**Affiliations:** 1 Sino-French Joint Laboratory for Invasive Forest Pests in Eurasia, Beijing Forestry University, Beijing 100083, China; 2 Beijing Key Laboratory for Forest Pest Control, Beijing Forestry University, Beijing 100083, China; 3 State Key Laboratory of Rice Biology, Institute of Insect Sciences, Zhejiang University, Hangzhou 310058, China

**Keywords:** invasive pests, climate change, CMIP6, MaxEnt, potential distribution

## Abstract

**Simple Summary:**

*Obolodiplosis robiniae* is an invasive species found across Eurasia. This species has now established itself as a common pest of black locust trees, with widespread infestations resulting in severe defoliation and substantial ecological and economic damage. Considering its biology and ecology, we applied a machine-learning algorithm based on the maximum entropy principle. The model’s predictions are consistent with the known distribution of *O. robiniae*. The total potential suitable area is 10,896,309.16 km^2^. In the context of future climate warming, the suitable area will extend to high latitudes, whereas the area in the south will decrease slightly. Governments should be concerned about the potential spread of *O. robiniae* throughout Eurasia, and quarantine measures should be implemented as soon as possible to avoid astronomical maintenance costs later on.

**Abstract:**

*Obolodiplosis robiniae* was discovered in Eurasia at the beginning of the 21st century. In this study, we explore the present and future (in the years 2050 and 2070) trends in the potential distribution of *O. robiniae* in Eurasia under diverse climate change scenarios based on a maximum entropy model. Our findings indicated that the current potential distribution area of *O. robiniae* is within the range of 21°34′ and 65°39′ N in the Eurasian continent. The primary factor controlling the distribution of *O. robiniae* is temperature. The highly and moderately suitable areas are mainly distributed in the semi-humid and semi-arid regions, which also happen to be the locations where the host black locust (*Robinia pseudoacacia* L.) grows at its fastest rate. The forecast of the potential distribution area of *O. robiniae* revealed that the species would benefit from global warming. The region suitable for the habitat of *O. robiniae* is characterized by a large-scale northward expansion trend and an increase in temperature. This information would help the forestry quarantine departments of Asian and European countries provide early warnings on the probable distribution areas of *O. robiniae* and provide a scientific basis for the prevention and control of *O. robiniae* spread and outbreaks.

## 1. Introduction

Due to the globalization of trade and rising tourist traffic between continents, the introduction and spread of new species have been increasing annually [1,2]. Invasive alien species (IAS) have the potential to cause significant negative consequences on human health, economic viability, and ecosystem function, in the region where they are introduced [3,4,5]. In fact, because of their negative effect on the environment, invasive species are predicted to be among the main drivers of global change [6]. Additionally, it is anticipated that the warming of the world’s climate will make an increasing number of formerly unsuitable places more suitable for habitation by such species [7,8]. Insects, in particular, are more harmful as invasive species and can spread more quickly and widely due to their high reproduction rate, abundance, remarkable physiological resilience to temperature extremes, and capacity to fly [9]. An earlier study found that 940 of the 1600 insects species studied were significantly impacted by global warming, including its effects on insect populations, insect development rates, range expansion of alien invasive insect species, migration of insects to high altitude areas, likelihood of insect outbreaks, and reduction in insect population diversity [10].

The locust gall midge, *Obolodiplosis robiniae* Haldeman (Diptera: Cecidomyiidae), is a Nearctic pest that resembles the adult *Anopheles* mosquito in terms of body color, size, and morphology [11]. *O. robiniae* is a monophagous species with specialized feeding on *Robinia* species, including *R. pseudoacacia* L, *R. hisqida* L, and *R. pseudoacacia* Aurea. The gregariously feeding larvae cause the margins of the leaflets to thicken and bend downwards, forming the characteristic leaf margin roll galls [12,13,14]. This insect harms the black locust (*Robinia pseudoacacia*) throughout the year, from the initial development of leaves to their fall, by hindering the leaf photosynthetic processes and causing early leaf abscission. Consequently, this leads to a decline in tree growth and productivity, thus affecting the economic, ecological, and ornamental value of the black locust. Additionally, these injuries aid in the attack of secondary pests such as longhorn beetles and jewel beetles, which ultimately result in the mortality of trees [15,16].

In the 19th century, *O. robiniae* was first described as *Cecidomyia robiniae* in Pennsylvania (USA) [17]. Its habitat was restricted to North America, and until its discovery in Asia in 2002 (in Japan and Korea) and then in Europe (Italy) in 2003, *O. robiniae* did not receive much attention [18,19,20,21]. In Europe, *O. robiniae* has been spreading faster than anticipated, with recent reports of infestations in 26 European nations [22]. Between 2003 and 2006, *O. robiniae* traveled over 2000 km east from Veneto, Italy, to Donetsk, Ukraine [23]. In 2007, it reportedly crossed the English Channel to reach Oxford, England (UK), in Western Europe [24]. A year later, in 2008, it was discovered in Sweden’s Lund in Northern Europe, indicating a tendency for *O. robiniae* to spread eastward [25]. In China, *O. robiniae* was first discovered in Qinhuangdao, Hebei Province, in 2004 [26]. Following that, an explosive spread occurred throughout China, and the species was detected in Northeast Liaoning in 2005 [27]; it also spread to Jilin, Beijing, and Shandong in 2006 [28,29,30]. Currently, it has been detected in 17 provinces (municipalities/autonomous regions) of China, which are broadly dispersed between 26°35′ and 43°58′ N and 103°48′ and 121°15′ E at an altitude of 8.05 m and 1561.33 m.

Zhang et al. used the CLIMEX model to predict and analyze the potential geographic distribution of *O. robiniae* in China. The quantitative analysis of the pest risk carried out by these researchers predicted that the risk value in China was 2.26 and represented a highly dangerous organism according to the International Plant Protection Convention (IPPC) of risk values [31]. Zhao et al. assessed the present and future (2050) changes in the areas suitable for *O. robiniae* habitat in China using the maximum entropy (MaxEnt) model. They discovered that the future habitat is bigger than the overall habitat range under the current climatic conditions, and the leading cause of this increase was the expansion of the highly and moderately suitable areas [32]. Due to limited prior research and scarce data on its actual spread, *O. robiniae* was considered to still be in a state of diffusion. In this study, we present our analysis of the distribution of *O. robiniae* in China, along with more complete occurrence records and updated global climatic data gathered from v1.4 to v2.1 of the WorldClim database in 2020 [33,34]. This provides an opportunity for updating and improving the prediction of the potential distribution range of *O. robiniae*.

In this study, we aimed to predict the current potential distribution (CPD) of *O. robiniae* in Eurasia and its future (years 2050 and 2070) potential distribution (FPD) change trend. To do this, we used the MaxEnt model, new climate data, and the results of our observations in China. We also identified the dominant climate variables influencing the distribution of *O. robiniae*. Our findings will serve as a crucial reference and guide for the existing and future control and quarantine of *O. robiniae* by forestry and customs quarantine authorities.

## 2. Materials and Methods

### 2.1. The Species Occurrence Data and Environmental Variables

We found 1008 occurrence records of *O. robiniae* in Eurasia: (1) all occurrence records collected in China originated from our field survey; 125 occurrence data of *O. robiniae* were recorded in 26 cities in 15 provinces (municipalities/autonomous regions); and (2) the data of the distribution points outside of China were obtained from the Global Biodiversity Information Facility (GBIF, https://www.gbif.org/, accessed on 1 March 2021) (648 records) and published in the literature statistics (236 records) (Figure S1).

The climatic data were downloaded from the WorldClim website (http://www.worldclim.org, accessed on 1 March 2021). The Global Climate Data Version 2.1 with a resolution of 2.5 min was employed. It contained a total of 19 bioclimatic variables (Table S1). Bioclimatic variables were derived from analyses of the annual trends and biologically significant parameters obtained from seasonal temperature and rainfall data values, which are essential for the species’ survival in a particular habitat. These climatic parameters were used in ecological studies to evaluate the effects of climatic conditions and their possible distribution [35,36].

In order to comprehensively evaluate the changes in the potential suitable regions of *O. robiniae* in the future periods, specifically years 2041–2060 (2050) and 2061–2080 (2070), we employed three different global climate models (GCMs): BCC-CSM2-MR, CNRM-CM6-1, and IPSL-CM6A-LR [37]. We used the GCMs from the CMIP6 of the sixth assessment report (AR6) of the Intergovernmental Panel on Climate Change (IPCC). Three Shared Socio-economic Pathways (SSPs) were selected for each of the GCMs: SSP126 [38], SSP370 [39], and SSP585 [40]. The three SSPs emission scenarios were then considered to represent a low-forcing, medium-forcing, and high-forcing scenario of climate change with economic development.

### 2.2. Optimization of the Model Parameter

The MaxEnt software is an ecological niche model based on environmental variable layers and species occurrence records. It integrates machine learning and the principle of maximum entropy to simulate the potential geographic distribution of species [41]. In order to reduce the sampling bias caused by oversampled areas, we calibrated the CPD for each species by filtering the specimen records to the spatial resolution of the environmental layers used (2.5 arc-minutes), resulting in one record per cell [42]. Through this approach, we obtained 659 points after filtering.

There exists a certain correlation among the 19 bioclimatic variables, and too many variables increase the dimensionality of the ecological niche, thereby affecting the prediction performance and precision of the MaxEnt model [43,44]. We next used the ArcMap version 10.3 (ESRI, Redlands, CA, USA) software to couple the 19 bioclimatic variables with 659 occurrence records to perform Pearson’s correlation analysis (Figure S2); we removed the climate layer with a low biological significance in the high-correlation variable group, and filtered out 10 environment layers (Table S1).

Additionally, we conducted our first exploratory analysis using the MaxEnt version 3.4.4 (results not shown) and the “ENMTools” package in R, version 4.0.5 (https://www.r-project.org/, accessed on 1 March 2021), to adjust and optimize the feature combination (FC) of the MaxEnt and regularization multiplier (RM) β parameters. As a result, we effectively reduced the model’s complexity and improved the fit between the predicted and actual results. The β multiplier settings of 0.1–5 and 0.5 gradually increased. The FC included linear (L), quadratic (Q), hinge (H), product (P), and threshold (T). We tested eight distinct FC combinations, including L, LQ, LQP, QHP, LQH, LQHP, QHPT, and LQHP [42,45,46], and obtained the corrected Akaike Information Criterion (AICc) of different parameter combinations. We then selected the minimum value of the AICc as the optimal setting and established the model (Figure S3a) [47].

In order to assess its predictive performance, the species occurrence records were utilized for model calibration by dividing them into a training set (75% of the total occurrence records) and a test set (25% of the total occurrence records). The relative probability calculated for each grid was used here as the relative habitat suitability of *O. robiniae*. To improve the prediction accuracy and lower the level of uncertainty, subsample validation was set up in the model, and 10-fold repetitions were performed to obtain average results. Then, the response curves and jackknife were created to measure the importance of the variables, removing climate variables with low contributions to obtain the final six climate variables (Table S1) [48].

In order to minimize model overfitting, Principal Component Analysis (PCA) of the climate variables was performed to estimate the heterogeneity of six bioclimatic variables using ArcMap software (ArcToolbox, SDM Tools) [49]. We aimed to ensure that the occurrence records were spatially independent, thereby reducing the over-fitting of the model to environmental biases. We then carried out spatial filtering based on the value of the environmental heterogeneity and finally obtained the occurrence records (Figure S1) [50].

### 2.3. Classification of CPD and FPD

A second construction of the model using the selected occurrence records and bioclimatic variables was used for the development of CPD maps and 18 FPD maps for the prediction of *O. robiniae* spread (Figures S4 and S5). To balance the differences between GCMs and present a trend for a higher likelihood of FPD occurrence [51], we averaged the results of these three GCMs for SSP126, SSP370, and SSP585, respectively, and finally obtained six FPD maps (years 2050 and 2070) for different SSPS scenarios of *O. robiniae* spread.

We used the lowest presence threshold (LPT) to define the suitable and non-suitable areas [52]. The potential area distribution was divided into four categories, including unsuitable (0-LPT), marginally suitable (LPT-0.4), moderately suitable (0.4–0.6), and highly suitable (0.6–1.0) areas. All layers were generated as binary maps using ArcMap software (ArcToolbox-Spatial Analyst Tools-Reclass-Reclassify) based on LPT thresholds, and then the functions of “Distribution Changes Between Binary SDMs” and “Centroid Changes (Lines)” of SDM Toolbox are used, respectively, to analyze the changes in the CPD and FPD areas and the centroid movement trend.

### 2.4. Assessing the Accuracy of the Model

The performance of the MaxEnt model was evaluated by the Receiver Operating Characteristic (ROC) method, and the area under the curve (AUC) was calculated as a measure of the prediction accuracy. The AUC values >0.5 imply a better-than-random fit, with 0.9 < AUC < 1 representing high predictive ability. However, using only the AUC value may be biased, so we also use the true skill statistic (TSS) and the AUC value of the partial-area ROC (P-ROC AUC) to estimate the accuracy of the MaxEnt model. TSS ranges from −1 to +1, where +1 indicates perfect agreement and ≤0 indicate a performance no better than random [35]. AUC ratios were calculated using Niche Analyst software with an error rate of E = 5%. AUC ratio = AUCE/AUC0.5. When the AUC ratio is greater than 1, the model has a high degree of credibility [53]. In addition, we used the minimum training presence omission rate (ORmtp) and 10 percentile training presence omission rates (OR10), with expected values of 0 and 0.1, respectively, to verify if the model was overfitting [54]. When the AUC value of the model is high and the omission rate is near the predicted value, the model has a strong predictive ability [41,55].

## 3. Results

### 3.1. The Main Parameters and Performance of the Model

The current model was constructed using 248 occurrence records of 6 bioclimatic variables, FC for LQHP, and RM of 0.5 (Figure S3c), and it showed very good performance (AUC = 0.954 ± 0.004, TSS = 0.830 ± 0.058, AUC Ratio = 1.635 ± 0.428, ORmtp = 0.011 ± 0.012, OR10 = 0.135 ± 0.035) (Table S2 and Figure S6) and an LPT of 0.043.

The most important bioclimatic variables that determined the potential distribution of *O. robiniae* were the Annual Mean Temperature (Bio1), Annual Precipitation (Bio12), and the Precipitation of the Driest Month (Bio14). The total contribution of these three climatic factors was 86.6% (Table 1). The response curve (Figure S7b–d) revealed that the climatic suitability of *O. robiniae* had a unimodal relationship with Bio1 and Bio12, while Bio14 had two peaks at 2.80 mm and 46.93 mm. For Bio1, the temperature range of the highly suitable for *O. robiniae* was 8.31 °C to 12.31 °C, with a standard deviation of 1.17 °C. For Bio12, the range of the highly suitable was 632.99 mm to 1392.57 mm, and the standard deviation of Bio14 was 18.5 mm. The future values of these three layers exceeded their current values, with a wider range and an average increase of 9.7% (Table S3). According to Jackknife (Figure S7a), a shorter green band signifies that the environmental variable has more information than other variables; in this case, the influence on the species distribution is greater. As can be seen in the figure, Bio1, Bio3, and Bio15 provided more information specific to the prediction of the distribution area of *O. robiniae* and were thus indispensable.

### 3.2. Predicted Current Potential Distribution

**Figure 1:**
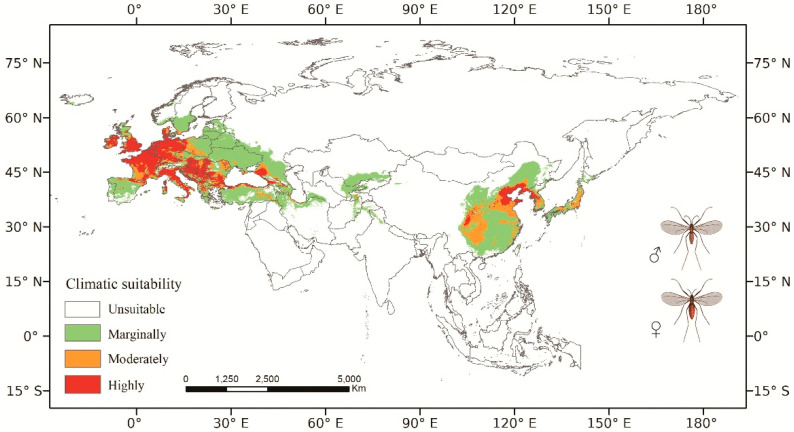
Prediction of the current potential distribution area of *O. robiniae* in Eurasia. Colors indicate the probability of climatic suitability: unsuitable area (0–0.043), marginally suitable area (0.043–0.4), moderately suitable area (0.4–0.6), and highly suitable area (0.6–1.0). Spatial resolution: 2.50 min; Geographic Coordinate System: WGS 84.

**Figure 2:**
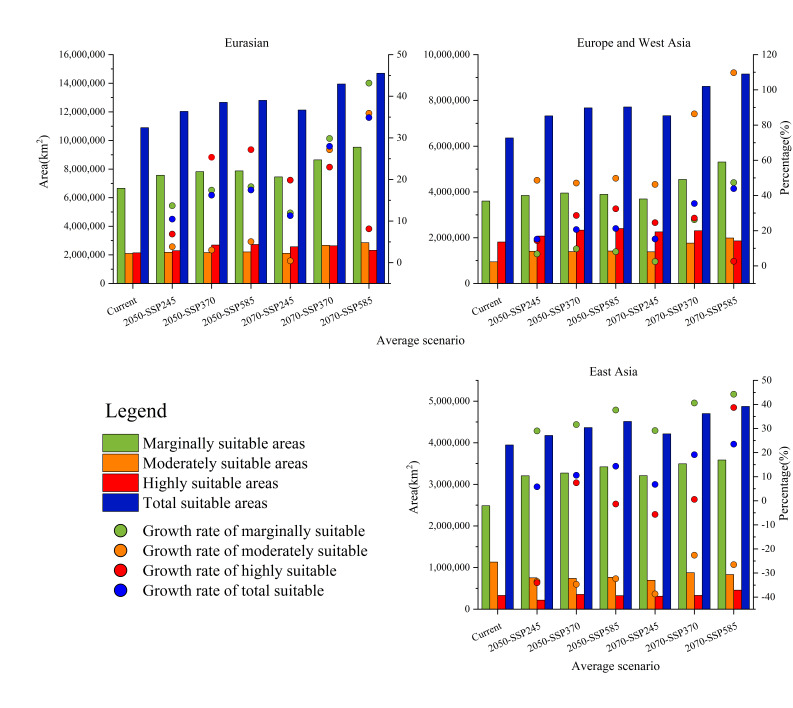
Predicted suitable areas for *O. robiniae* under present and future climatic conditions (km^2^) and percentage (%) of increase/decrease (compared to the currently suitable areas).

The total CPD area of *O. robiniae* in Eurasia was 10,896,309.16 km^2^ (Figure 1 and Figure 2), with suitable areas covering a substantial portion of Europe. The moderately and highly suitable regions (orange and red) were predominant, covering 38.91 % of the total area of Europe. These regions included the majority of Western Europe (the central and southern parts of the United Kingdom, the north of Spain, France, Germany, etc.), the southwestern region of Central Europe (southwestern Poland, Hungary, etc.), the north of Southern Europe (the south of Romania, the north of Greece, Serbia, etc.), a small part of Northern Europe (Denmark and south of Sweden), and Eastern Europe, mainly the plains on the east side of the Black Sea. In Asia, Turkey and northern Iran in West Asia, and southern Kazakhstan and eastern Uzbekistan in Central Asia were suitable areas, but with a relatively low degree of fitness. The highly and moderately suitable regions were distributed mainly in East Asia, the eastern part of Japan, the southwest of the Korean Peninsula, and in northeast China (Liaoning Province), north China (Beijing, Tianjin, Hebei, Shandong, etc.), and southwest China (Guizhou Province, Sichuan Province, etc.). The *O. robiniae* suitable area in West Asia was closely associated with that in Europe. In order to assist our research and minimize the fragmentation of the suitable areas, our subsequent study divides the potential distribution areas into two categories. The suitable areas in Europe and Western Asia (EWA) are placed in one category, and the other comprises East Asian countries.

### 3.3. Predicted Future Potential Distribution and Analyzed Tendencies

**Figure 3:**
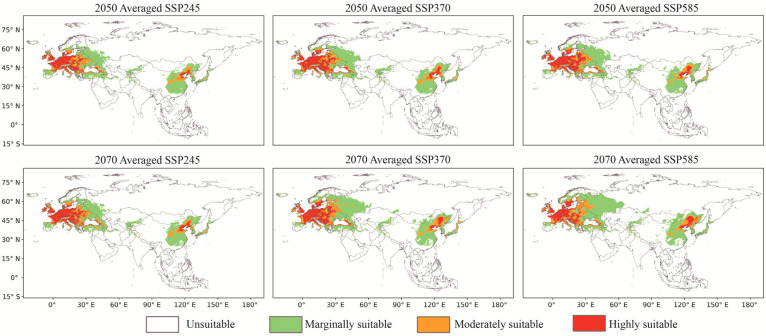
Averaged projected ranges of *O. robiniae* invasion for three climate change scenarios: SSP245, SSP370, and SSP585 for 2050 and 2070, SSPs = shared socio-economic pathways.

The outcomes of the MaxEnt model obtained under the future climate change scenarios SSP126, SSP370, and SSP585 for 2050 and 2070 are presented in Figure 3. It is evident that, compared with CPD, in the same year of FPD, the total suitable area of *O. robiniae* increases with the increase in the radiative force in the SSPs emission scenario. In the same SSPs emission scenario, the suitable area in 2070 would also be larger than that in 2050 (Figure 2). The marginally suitable area occupied the major part, followed by the highly suitable area. With the exception of 2070-SSP370 and 2070-SSP585, the moderately suitable area was larger than the highly suitable area. The largest FPD area was present under the SSP585 scenario in 2070. The total area was 34.87% larger than that of CPD, covering 14,696,253.77 km^2^, mainly due to the expansion of the marginally and moderately suitable areas, which increased by 43.15% and 35.91%, respectively. The moderately and highly suitable areas for *O. robiniae* habitats would continue to spread northwards in Eurasia in the future, as can be seen in Figure 3. In EWA, the west coast of Norway, Poland, the central and western parts of Ukraine, and the southern part of Belarus and Sweden would become the new areas severely affected by the spread of *O. robiniae*. In East Asia, Japan and South Korea would gradually develop into marginally suitable areas for *O. robiniae* habitats. In China, the moderately and highly suitable areas in the southwest would shrink progressively, and the three provinces in the northeast would gradually become the worst-hit areas. Although the CPD of *O. robiniae* generally tended to spread northwards, subtle variations existed between the EWA and East Asia. In EWA, the expansion of the moderately and highly suitable areas was predominant, whereas in East Asia, the growth of the marginally suitable sites was dominant, and the moderately suitable areas were diminishing.

**Figure 4:**
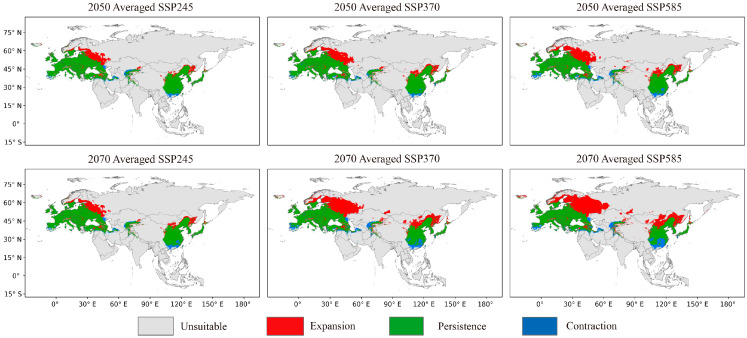
Shift in the *O. robiniae* average potential distribution range under the three climate change scenarios for 2050 (2041–2060) and 2070 (2061–2080), compared with the current potential distribution (green corresponds to areas of persistence, blue of the contraction zone, and the red is the expansion zone). Spatial resolution: 2.50 min.

**Figure 5:**
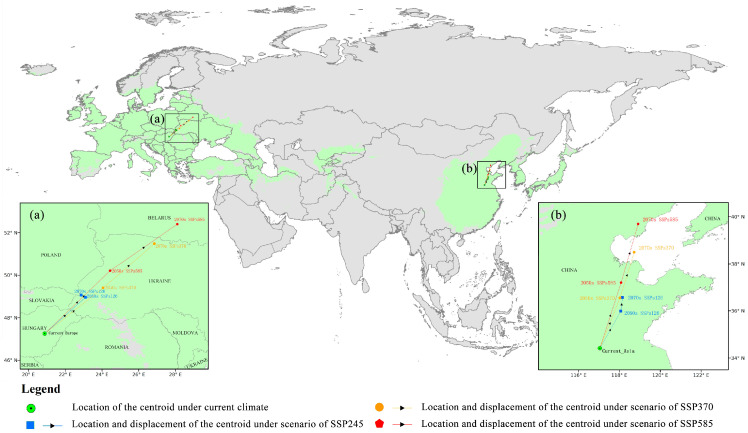
Map of centroids range shifts depicts the predicted future distribution changes under each scenario. Each line depicts predicted distributional shifts of *O. robiniae* range centroid from current (start of the arrow) to 2070 (end of the arrow) scenarios. (**a**) Centroid change in Europe (**b**) Centroid change in East Asia. Green represents the current potential distribution of *O.robiniae*, while a green circle represents the current centroid.

Figure 4 illustrates the trends in the expansion and contraction of CFD of *O. robiniae*, primarily highlighting large-scale expansion in the north (red) but a small-scale reduction in the south (blue). The central and southern parts of Spain, as well as the majority of southern China, would eventually become non-suitable areas. However, regions currently unsuitable for *O. robiniae*, such as the European Alps, the Taurus Mountains in West Asia, and the Hexi Corridor in China, would eventually become more suitable. Combined with the trend of centroid change (Figure 5), it can be seen that the centroid of FPD in East Asia and EWA relocated to the northeast, whereas the centroid of East Asia was adjusted to the northeast of China (azimuth angle 20.03 ± 7.6°), and the centroid in Europe changed at a larger angle, mainly towards central Russia (azimuth angle 40.10 ± 4.74°). With the enhancement of the SSPs emission scenarios, the FPD centroid offset distance was also increasing. The farthest EWA SSP585 scenario in 2070 was 773.81 km from the current centroid. At that time, the *O. robiniae* suitable area would cross the Ural Mountains, reaching the West Siberian Plain.

## 4. Discussion

The assessment and forecasting of the impact of invasive alien species on a global scale have drawn a lot of research interest [35,36]. *O. robiniae*, a native of the Nearctic region, invaded almost simultaneously from the eastern and western parts of Eurasia at the beginning of the 21st century, and it then spread rapidly, thanks in large part to the belief that it did not pose a serious threat to forest stands, frequent trade activities, and the widespread presence of its host *R. pseudoacacia* [11,37]. However, our investigation revealed that human negligence was the primary cause for its spread in almost every region inhabited by *R. pseudoacacia* in China. *R. pseudoacacia* is found in the coastal protective and timber forests and on other forest lands and is also extensively distributed in urban areas such as university campuses, wetland parks, and residential neighborhoods. *O. robiniae* is illegally reproduced in China. A compound leaf can contain a maximum of 54 galls, and a maximum of 18 larvae can feed gregariously within each gall, resulting in damage rates of up to 90–100% in July and August [27,38,39]. The infestation of *O. robiniae*, which perms the crown of black locusts into “curls”, has detrimental ecological and socio-economic effects. Moreover, its distribution range continues to grow further, reaching the city of Lanzhou in 2017 (It had previously spread only to Tianshui in western China). Although *O. robiniae* is a newly introduced species, it is already a common insect because of its unique galls and exclusive host features, which facilitate its easy and accurate identification; it can be easily located by careful observation of the eaves of black locust [40]. It is essential to identify the key climatic factors influencing the distribution of *O. robiniae* and to predict the potential distribution area, to prevent the further spread of *O. robiniae*. The MaxEnt software offers a high level of reliability as a model for predicting the potential distribution area of species [41].

### 4.1. Analysis of Model Results and CPD

We completed the first study of the potential range of *O. robiniae* distribution in Eurasia using the MaxEnt software, the literature evidence, data obtained from GBIF, and a comprehensive field survey in China. Through relevant screening of the available occurrence records, optimization of bioclimatic variables, and adjustment of model parameters, the performance of the present MaxEnt model was confirmed to be good; the ROC curve, TSS and omission rates (Figure S6) objectively corroborated these results.

In this study, the suitable and non-suitable areas were defined by the LPT, indicating that the CPD of *O. robiniae* was distributed between 21°34′ and 48°03′ N in East Asia and between 34°57′ and 65°39′ N in Europe. In predicting the potential range of Diptera, Annual mean temperature (bio1) is often used as an important driving force for the distribution of species, such as *Carpomya pardalina* [42] and *Anastrepha grandis* [43], and our results are consistent. The results demonstrated that the distribution of *O. robiniae* was strongly driven by three factors: Annual Mean Temperature (Bio1), Annual Precipitation (Bio12), and Precipitation of the Driest Month (Bio14). The most suitable climate for *O. robiniae* breeding is under the following main factor values: Bio1 = 9.42 °C, Bio12 = 798.15 mm, and Bio14 = 46.94 mm (Figure S7b–d). These findings were consistent with the data from our survey in China, where *O. robiniae* was severely prevalent. In the survey, we found that not only did *O. robiniae* cause severe damage in semi-humid, semi-arid, and warm areas, but also that the number of larvae in a single gall was significantly higher. These regions overlapped heavily with the moderately and highly suitable areas of the CPD of *O. robiniae* and had excellent germination and tillering capacities for the host black locust. These areas also provided facilities and opportunities for the prolific expansion of *O. robiniae* because the adults only lay eggs on newly sprouted leaves.

Although we used the most recent climate data collected from WorldClim Version 2.1, compared to the current period (2021) released at the time of manuscript preparation, there was still a lack of 20-year data, and a remarkable trend in the climate warming is observed for the period from 2000 to the present time [44,45], so the predictive result of the CPD of *O. robiniae* is rather conservative. Additionally, in many countries, the host black locust is extensively planted in areas such as parks, by the sides of roads, and other places significantly impacted by human management. For instance, we found planted black locusts in residential quarters and parks in Turpan, Xinjiang, China. A microclimate favorable for its growth was created through human management, which promoted the growth of these black locust trees. This unique artificial microclimate [46], combined with the biological characteristics of *O. robiniae* larvae, which overwinter as cocoons in the soil and have a supercooling point of −12.19 °C (data not published), would unquestionably aid their successful future establishment and spread in Turpan. The two aforementioned observations suggested that the suitable area of *O. robiniae* in Eurasia was more expansive than the predicted CPD range, especially the northern boundary of the suitable area, which will soon be breached.

### 4.2. Analysis of the Trends of the FPD of O. robiniae

In the 2050s and 2070s, along with global climate warming, the FPD of *O. robiniae* would continue to expand on the Eurasian continent, mainly to the north. The average suitable area would increase by 14.72 ± 4.26% in 2050 compared to the CPD area, and by 8.56 ± 7.81% in 2070 compared to the area in 2050, dominated by an increase in the marginally and moderately suitable areas. These results demonstrate that *O. robiniae* benefits from global warming. Although the southern regions of China and Spain would no longer be suitable for *O. robiniae* due to high temperatures and humidity changes in the future, the reduced area is still much smaller than the increased area to the north (Figure 4), with the largest expansion in EWA, averaging 1,607,771.89 km^2^, 3.23 times larger than the area of growth in East Asia. Similar outcomes were obtained in the shift movement of the centroids. The average moving distance of centroids in EWA in 2070 was predicted to be 554.98 km, 23.77% greater than the offset distance of centroids in East Asia. Currently, *O. robiniae* has extensively colonized the black locust planting area on the Eurasian continent, and its FPD expansion trend is well synchronized with the FPD expansion trend of black locusts in Europe [47], creating a favorable environment for the rapid spread of *O. robiniae* in the future.

The impact of climate change on species distribution is becoming increasingly significant. IPCC AR6 has performed a large number of scientific assessments and concluded that the recent global warming has been more widespread, faster, and more intense than that witnessed for thousands of years [48]. Studies have shown that changes in FPD trends may appear sooner than previously expected. Black locust, for example, appears to have a strong capacity for climatic adaptation, and its potential distribution in Eastern Europe may increase 20 years earlier than initially anticipated [49]. Although we did not find any infestation of *O. robiniae* in Heilongjiang Province, China, during our survey (Changchun, Jilin Province, has the northernmost occurrence records in China), we determined that the area’s semi-humid environment may be conducive to *O. robiniae* colonization, which also necessitates routine monitoring of the margins where *O. robiniae* has been recorded to occur. The likelihood is that it has already spread 200 km outwards this year.

### 4.3. Final Considerations and Quarantine Management Measures

Even though many species can spread independently, anthropogenic factors play a role in biological invasion, and long-distance dispersal is often triggered by or connected to human activity [50]. For example, motorway networks have a role in the distribution of *O. robiniae*. The long-distance transmission of *O. robiniae* is caused mainly by the movement of black locust larvae, and the adults or infected leaves may be disseminated by the wheels and other components of motor vehicles [51]. In our investigation, we found that the numbers of *O. robiniae* individuals in cities and villages were not significantly different [46], but abundant pest occurrence was observed on isolated hills or small islands in the middle of lakes, where the wind may have played a major role given the frail body of adults [52]. This spread was also closely related to the exponential population explosion during the growing season and the large-scale cultivation of black locusts.

Currently, the natural enemies of *O. robiniae* include predatory insects such as lacewings, ladybeetles, and crickets [37,53], as well as parasitic insects such as *Platygaster robiniae* Buhl and Duso, *Eupelmus urozonus* Dalman, and *Mesopolobus mediterraneus* Mayr, [37,54], with *P. robiniae* occupying a dominant position in parasitic wasps. It has been detected in European and East Asian countries, where it is considered to be one of the key contributors lowering the *O. robiniae* population density [55,56,57]. However, the outbreak period of *P. robiniae* lags behind that of *O. robiniae*, and its influence on population decline begins primarily with the third generation of *O. robiniae* and continues thereafter. Even when *O. robiniae* is parasitized, parasitic wasps will delay the development stage, remaining in a prolonged egg stage or embryonic period until the host larva has almost fully grown, and would not stop the leaves from curling and forming galls; as a result, an ideal control effect cannot be achieved [58]. We believe that human intervention is necessary in addition to biological control to prevent the spread of *O. robiniae.* Due to the protection of *O. robiniae* by the gall during the vegetation period and the high reproductive capacity of females, early prevention and control can be more effective as management strategies. Therefore, winter and spring are the key periods when prevention and control should be realized. After the leaf fall of black locust at the end of October, the fallen leaves should be promptly cleaned, burned, and buried to prevent larvae overwintering. In spring, the leaf-expansion period of black locust coincides with the peak period of overwintering adult emergence. Systemic insecticide sprays are also an effective approach against the spread of the pest. The main strategies for pest management are the control of the total occurrence of larvae, achieving a reduction in the population density and the occurrence base of pests, and obtaining one-time protection ensuring no harm throughout the year [29,59].

Climate warming causes early spring phenology of plants in most parts of the world, such as leaf bud opening, leaf spreading, and flowering, which further affects predator activities [60,61]. *O. robiniae* is a beneficiary in this respect. It is an adaptive multivoltine insect whose number of generations changes with the changes in temperature and host. In Europe, a number of 2–4 generations per year is typical, whereas 7 generations per year were noted in the Lunan region of China. As the climate warms, the abundance and destructive activities of *O. robiniae* will increase, reducing the growth rate of black locusts in large areas [62,63]. Although it appears that *O. robiniae* has not yet acquired the status of a pest, this status could alter in the future. Therefore, its potential occurrence in the future and economic significance should be closely monitored. The rapid spread of *O. robiniae* is due to its fast reproduction and widespread distribution of its host. Its control or eradication after its invasion and spread have begun is extremely difficult and expensive [64]. As a result, it is strongly recommended that, based on the predicted FPD trend of *O. robiniae* distribution, appropriate control measures should be swiftly implemented to prevent its further spread.

## Figures and Tables

**Table 1:** MaxEnt results of the percentage contribution and permutation importance of Bioclimatic variables for the selected model developed for *O. robiniae*.

Code	Environmental Variables	Percent Contribution
Bio1	Annual Mean Temperature	59.2
Bio12	Annual Precipitation	14.3
Bio14	Precipitation of Driest Month	13.1
Bio3	Isothermality	5.5
Bio15	Precipitation Seasonality	4.9
Bio6	Min. Temperature of Coldest Month	3.0

## Data Availability

The Global Climate Data Version 2.1 dataset was acquired from https://www.worldclim.org/data/index.html (accessed on 1 March 2021). The data used in this study are available at https://zenodo.org/record/6326587#.YiGCwMiKvZs (accessed on 4 March 2022).

## Supplementary Materials

The following supporting information can be downloaded at: https://www.mdpi.com/article/10.3390/insects14010048/s1, Figure S1: Current occurrence records and the map of Climate heterogeneity; Figure S2: Pearson correlation coefficient; Figure S3: Performances of ecological niche model; Figure S4: Potential suitable area in 2050; Figure S5: Potential suitable area in 2070; Figure S6: Accuracy evaluation of model prediction; Figure S7: Bioclimatic variables analysis; Table S1: List of bioclimatic environmental variables; Table S2: The predictive accuracy; Table S3: Summary of values of bioclimatic variables.
