# Supplementary material for: Future Trends in Obolodiplosis robiniae Distribution across Eurasian Continent under Global Climate Change
Source: Insects. 2023 Jan 3;14(1):48. doi: 10.3390/insects14010048 (PMC9861275; doi:10.3390/insects14010048)
Supplement: Supplementary file 1 [file insects-14-00048-s001.zip › insects-2103442-supplementary.pdf]

TableS1. List of bioclimatic environmental variables used to predict the Eurasian distribution of *O. robiniae*. The percentage contribution rate is the result of the first exploratory analysis. The bioclimatic variables were derived from long–term monthly temperature and precipitation (1970-2000). Spatial resolution: 2.50 minutes.

Code	Bioclimatic variables	Unit	Percentage contribution
BIO1 [‡]	Annual Mean Temperature	℃	41.2
BIO2[2]	Mean Diurnal Range (Mean of monthly (max temp - min temp))	℃	0.5
BIO3[‡]	Isothermality (BIO2/BIO7) (* 100)	%	2.9
BIO4[1]	Temperature Seasonality (standard deviation *100)	℃	
BIO5[2]	Max Temperature of Warmest Month	℃	0.9
BIO6[‡]	Min Temperature of Coldest Month	℃	15.1
BIO7[1]	Temperature Annual Range (BIO5-BIO6)	℃	
BIO8[1]	Mean Temperature of Wettest Quarter	℃	
BIO9[1]	Mean Temperature of Driest Quarter	℃	
BIO10[1]	Mean Temperature of Warmest Quarter	℃	
BIO11[1]	Mean Temperature of Coldest Quarter	℃	
BIO12[‡]	Annual Precipitation	mm/yr	10
BIO13[2]	Precipitation of Wettest Month	mm/mo	0.7
BIO14[‡]	Precipitation of Driest Month	mm/mo	15
BIO15[‡]	Precipitation Seasonality (Coefficient of Variation)	-	13.7
BIO16[1]	Precipitation of Wettest Quarter	mm/3mo	
BIO17[1]	Precipitation of Driest Quarter	mm/3mo	
BIO18[1]	Precipitation of Warmest Quarter	mm/3mo	
BIO19[1]	Precipitation of Coldest Quarter	mm/3mo	

Note: [1] Eleven variables were eliminated based on cluster and correlation analyses. [2]The three variables with low contribution were eliminated according to the first exploratory analysis. [‡]Final Six variables were selected for modeling

Table S2 The predictive accuracy of the maximum entropy model estimated by AUC, AUC ratio, ORmtp and OR10

Time Frame	SSPs	GCMs	AUC	AUC Ratio	ORmtp	OR10
Current	——	——	0.952	1.791	0.021	0.152
2050	126	BCC-CSM2-MR	0.953	1.755	0.005	0.121
2050	126	CNRM-CM6-1	0.953	1.728	0.013	0.140
2050	126	IPSL-CM6A-LR	0.954	1.207	0.016	0.147
2050	370	BCC-CSM2-MR	0.955	1.735	0.005	0.123
2050	370	CNRM-CM6-1	0.950	1.732	0.011	0.169
2050	370	IPSL-CM6A-LR	0.951	1.696	0.011	0.136
2050	585	BCC-CSM2-MR	0.953	1.207	0.008	0.148
2050	585	CNRM-CM6-1	0.951	1.717	0.016	0.170
2050	585	IPSL-CM6A-LR	0.958	1.664	0.008	0.107
2070	126	BCC-CSM2-MR	0.955	1.744	0.007	0.119
2070	126	CNRM-CM6-1	0.954	1.737	0.023	0.148
2070	126	IPSL-CM6A-LR	0.954	1.696	0.013	0.137
2070	370	BCC-CSM2-MR	0.953	1.640	0.013	0.123
2070	370	CNRM-CM6-1	0.953	1.659	0.005	0.134
2070	370	IPSL-CM6A-LR	0.953	1.591	0.005	0.145
2070	585	BCC-CSM2-MR	0.958	1.608	0.007	0.100
2070	585	CNRM-CM6-1	0.954	1.650	0.005	0.131
2070	585	IPSL-CM6A-LR	0.953	1.512	0.011	0.123

Table S3 Summary of values of bioclimatic variables used in the study for current conditions and prediction for 2050 and 2070 for each SSPs （the average of three GCMs）

Code	Current		2050						2070					
			SSP126		SSP370		SSP585		SSP126		SSP370		SSP585	
	Min	Max	Min	Max	Min	Max	Min	Max	Min	Max	Min	Max	Min	Max
bio1	-20.96	30.86	-18.31	32.62	-17.40	33.03	-16.69	33.44	-18.03	32.67	-15.55	34.17	-14.62	34.91
bio3	11.34	100.00	-1.10	95.34	-6.61	96.17	-7.45	95.07	-6.24	95.05	-13.05	94.62	-17.27	94.81
bio6	-49.90	26.20	-47.13	27.40	-45.53	27.77	-44.30	27.97	-46.40	27.47	-43.13	28.40	-41.63	28.90
bio12	0.00	8626.00	0.00	8692.67	0.00	8776.33	0.00	9266.00	0.00	8867.67	0.00	8872.00	0.00	9645.67
bio14	0.00	428.00	0.00	435.88	0.00	459.08	0.00	448.13	0.00	455.78	0.00	469.67	0.00	450.39
bio15	0.00	173.79	0.00	179.05	0.00	176.39	0.00	181.34	0.00	180.70	0.00	178.26	0.00	179.50

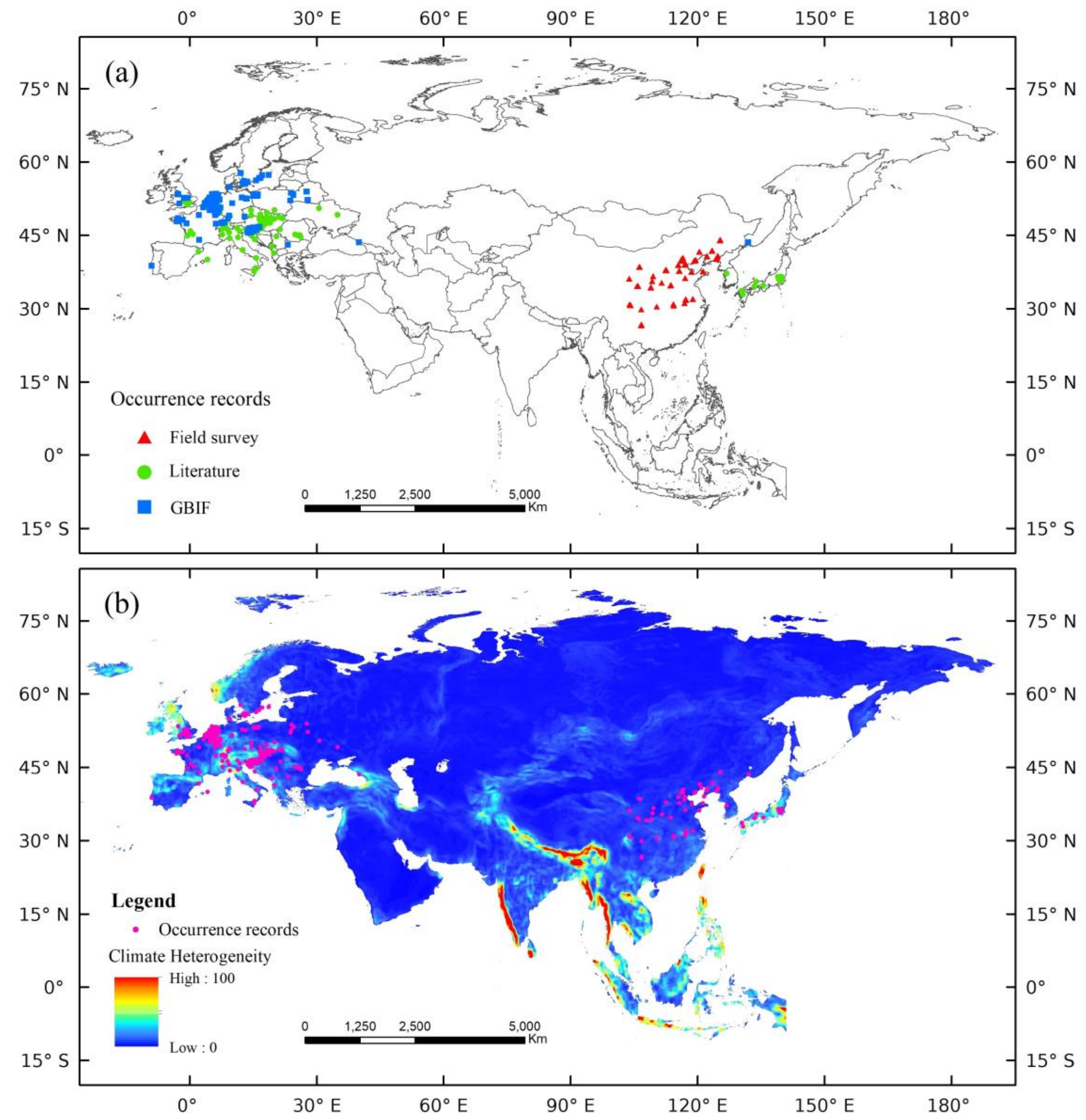

Figure S1. (a) Current occurrence records of *Obolodiplosis robiniae* in Eurasian obtained from the field survey（China）, literature and GBIF database. (b) The map of Climate heterogeneity was constructed using the final six bioclimatic variables, eliminating spatial clusters of localities to obtain the final occurrence records.

GBIF = Global Biodiversity Information Facility.

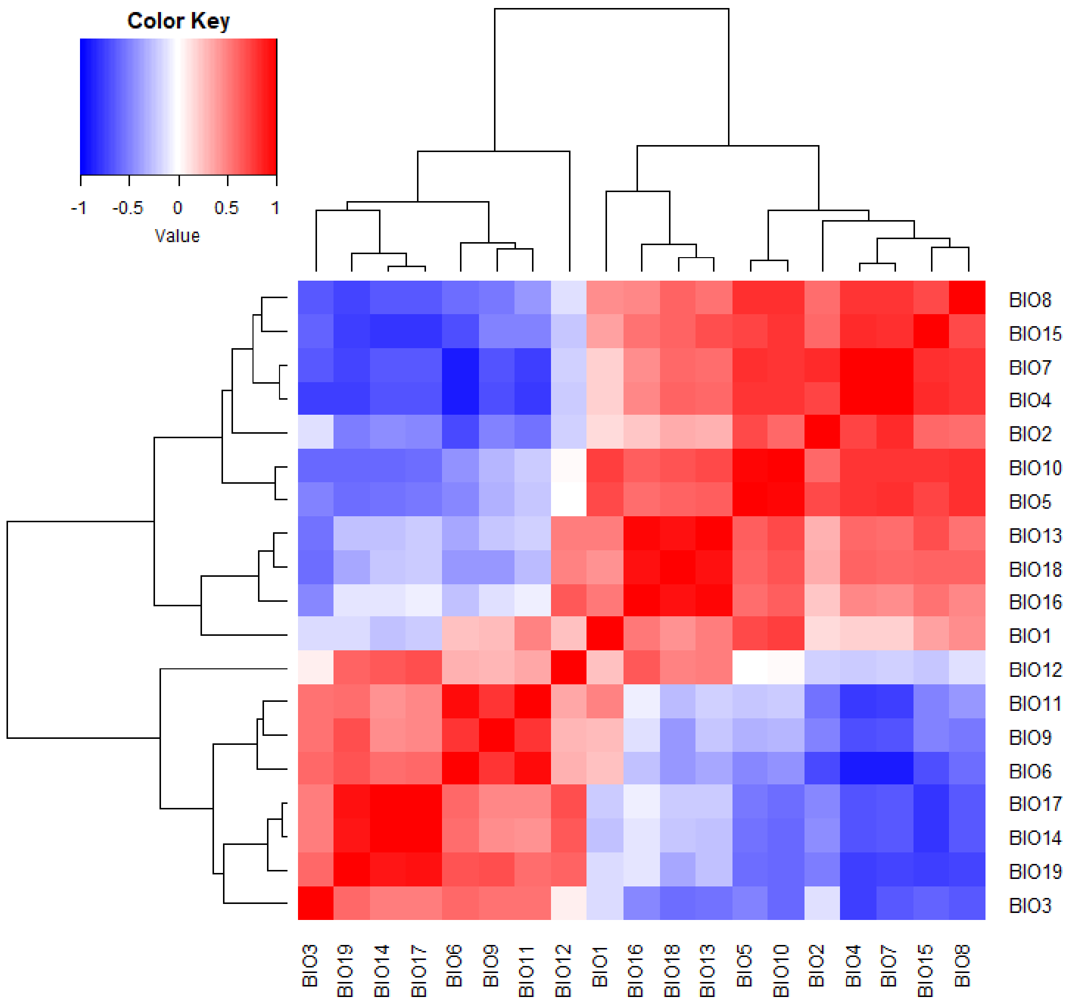

Figure S2. Hierarchical cluster analysis and Pearson correlation analysis of 19 bioclimatic variables . Created in R 4.0.5 (https://www.r-project.org/).

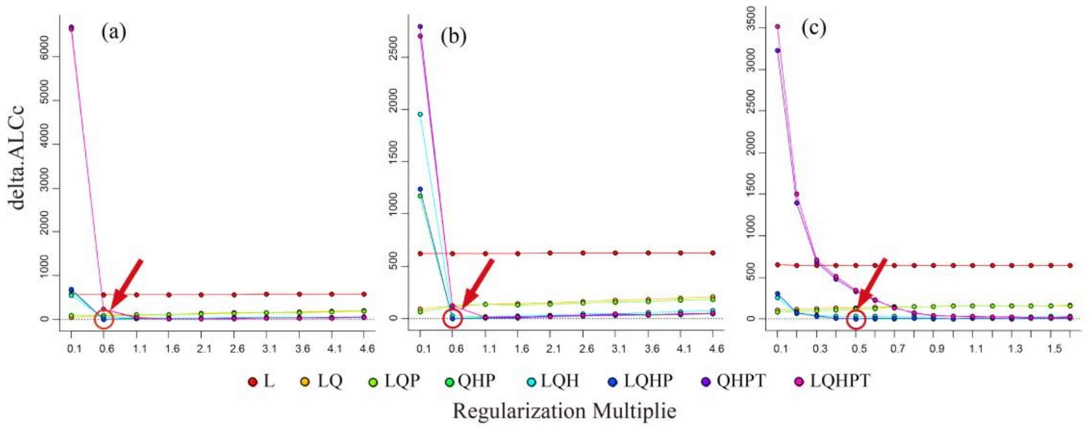

Figure S3. Performances of ecological niche model of *O. robiniae* have under different settings. Red arrow indicates the AICc chosen setting.

a: The result of the first exploration shows that the optimal parameter for FC is QHP and RM is 0.6. Eight FC combinations, β multiplier settings from 0.1 to 5,0.5 increments. b: For the second modelling, the optimal parameters are FC is QHP and RM is 0.6. Eight FC combinations, β multiplier settings from 0.1 to 5,0.5 increments. c: For further subdivision of the second modeling result, the best parameters are FC is LQHP and RM is 0.5. Eight FC combinations, β multiplier settings from 0.1 to 1.6,0.1 increments. β: Regularization multiplier; FC: feature combination, L-linear, Q-quadratic, H-hinge, P-product, T-threshold

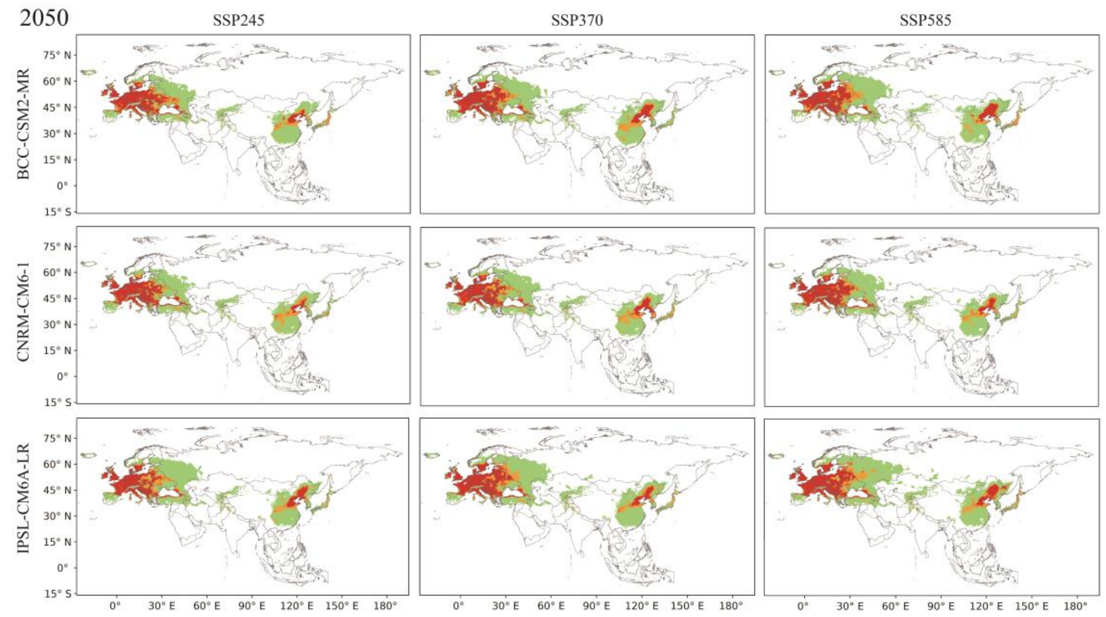

Figure S4. Potential suitable area of *O. robiniae* in 2050 according to MaxEnt models for three climate change scenarios: SSP245, SSP370 and SSP585 and three global circulation models (GCMs):BCC-CSM2-MR, CNRM -CM6-1, IPSL-CM6A-LR. Spatial resolution: 2.50 minutes.

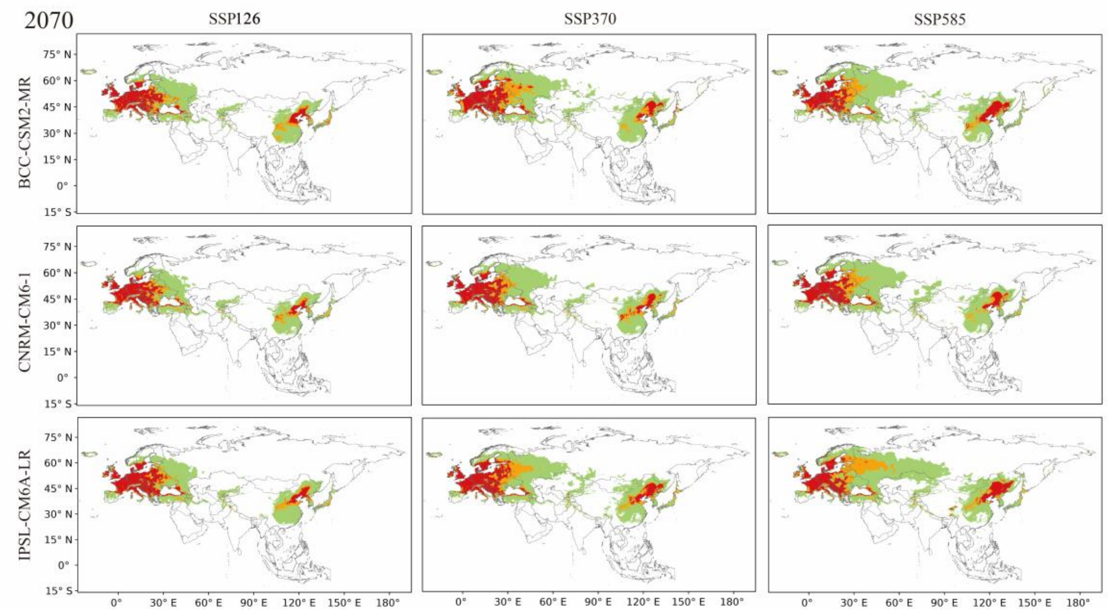

Figure S5. Potential suitable area of *O. robiniae* in 2070 according to MaxEnt models for three climate change scenarios: SSP245, SSP370 and SSP585 and three GCMs – BCC-CSM2-MR, CNRM -CM6-1, IPSL-CM6A-LR. Spatial resolution: 2.50 minutes.

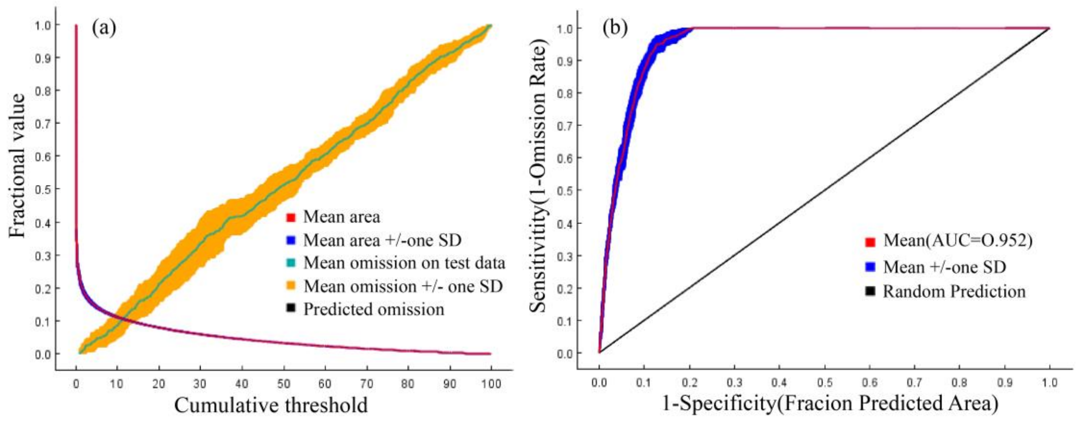

Figure S6. Accuracy evaluation of model prediction, (a) Average omission rate and predicted area as a function of the cumulative threshold, (b) Receiver operating characteristic (ROC) curve and AUC values under the current climate condition (10 replicated runs). The red curve indicates training data, the blue curve indicates test data, and the black line indicates random prediction, AUC, area under the curve.

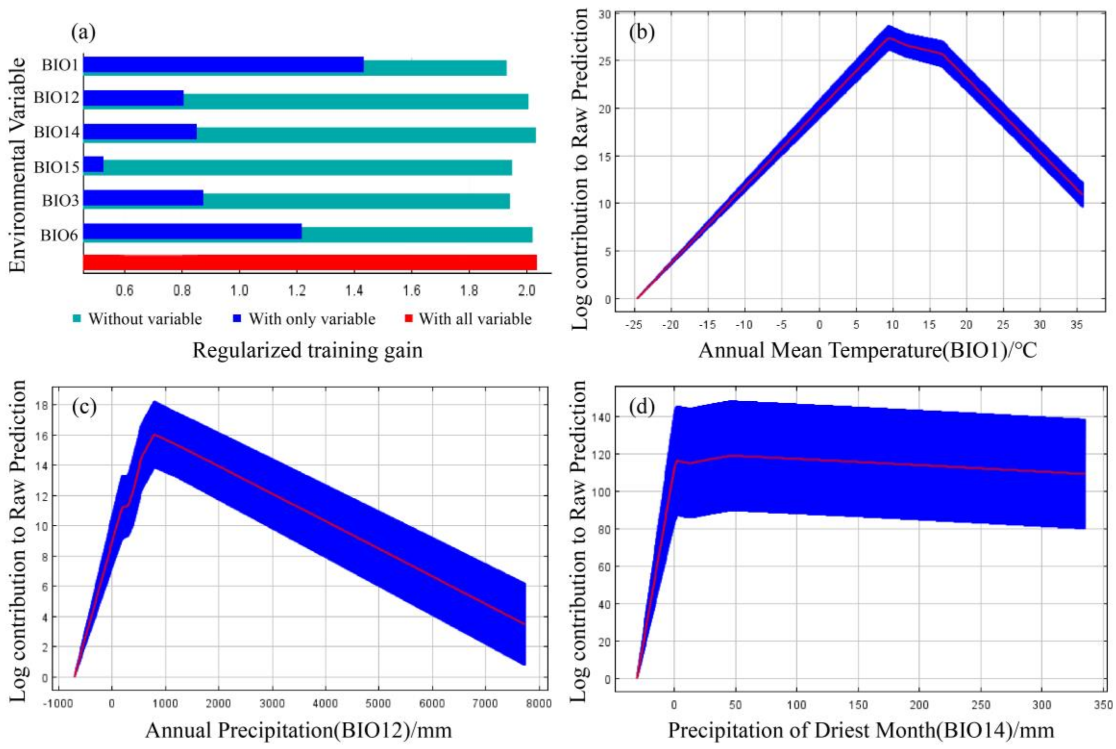

Figure S7. Bioclimatic variables analysis, (a) Jackknife of regularized training gain in MaxEnt models for *O.robiniae*, (b-d) Response curves of *O. robiniae* to bioclimatic variables with the highest contribution to model building. Red lines and blue areas show the average and standard deviation calculated over 10 replicates.
